# Host strain specific sex pheromone variation in *Spodoptera frugiperda*

**DOI:** 10.1186/1742-9994-5-20

**Published:** 2008-12-25

**Authors:** Astrid T Groot, Melanie Marr, Gerhard Schöfl, Sybille Lorenz, Ales Svatos, David G Heckel

**Affiliations:** 1 Max Planck Institute for Chemical Ecology, Dept. Entomology, Hans-Knöll Strasse 8, 07745 Jena, Germany; 2 Max Planck Institute for Chemical Ecology, Research group Mass spectrometry, Hans-Knöll Strasse 8, 07745 Jena, Germany

## Abstract

**Background:**

The fall armyworm *Spodoptera frugiperda *(Lepidoptera; Noctuidae) consists of two distinct strains with different host plant preferences for corn and rice. To assess whether pheromonal-mediated behavioral isolation accompanies the habitat isolation on different host plants, we compared the sex pheromone composition among females of the two strains. Pheromone glands were extracted with or without injection of pheromone biosynthesis activating neuropeptide (PBAN). To assess the mode of inheritance of this variation, we also analyzed the pheromone composition of F_1 _hybrid females.

**Results:**

Relative to intra-strain variation, the pheromone composition of the two strains differed significantly. Corn strain females contained significantly more of the second most abundant pheromone compound Z11-16:Ac (m), and significantly less of most other compounds, than rice strain females. When females were injected with PBAN before their glands were extracted, the differences between the strains were less pronounced but still statistically significant. The pheromone composition of hybrid females showed a maternal inheritance of the major component Z9-14:Ac (M) as well as of Z11-16:Ac (m). Most other compounds showed an inheritance indicating genetic dominance of the corn strain. The within-strain phenotypic correlations among the various components were consistent with their hypothesized biosynthetic pathway, and between-strain differences in the correlation structure suggested candidate genes that may explain the pheromone differences between the two strains. These include Δ9- and Δ11 desaturases, and possibly also a Δ7-desaturase, although the latter has not been identified in insects so far.

**Conclusion:**

The two host strains of *S. frugiperda *produce systematically differing female sex pheromone blends. Previously-documented geographic variation in the sexual communication of this species did not take strain identity into account, and thus may be partly explained by different strain occurrence in different regions. The finding of pheromone differences reinforces the possibility of incipient reproductive isolation among these strains, previously shown to differ in the timing of nocturnal mating activity and host plant use. Finding the genetic basis of the pheromone differences, as well as these other biological traits, will help to elucidate the role of premating isolation in the continuing differentiation of these two strains that may eventually lead to speciation.

## Background

In night-flying moths, highly specific, long distance, pheromonal communication ensures that males and females can find each other and mate. Females produce a species-specific sex pheromone in a specialized gland at the tip of their abdomen, to which males of the same species are attracted [1,2]. Moth pheromones usually consist of a blend of two or more components of even-numbered C_10_–C_18 _straight-chain, unsaturated derivatives of fatty acids, with the carbonyl carbon modified to form an oxygen-containing functional group (alcohol, aldehyde, or acetate ester) [3,4]. The species-specificity of each blend is determined by the particular combination of the components, as well as their relative ratios (e.g., [5-7]). Although there are thousands of moth species with unique pheromone blends (e.g., [8]), the evolutionary processes that resulted in this diversity of sexual communication signals are still poorly understood (e.g., [9-11]). To gain insight into the evolution of premating isolation between species it is essential to quantify the level and possible causes of variation in the premating signals within species on which selection may operate.

The fall armyworm (FAW, *Spodoptera frugiperda *J. E. Smith) offers an ideal opportunity for investigating intraspecific variation in sex pheromone communication, because two sympatrically occurring strains have already been recognized [12,13]. One feeds predominantly on corn (the corn strain, C) and the other on rice and various pasture grasses (the rice strain, R). These strains can be identified by several molecular markers, e.g. allele frequency differences at three allozyme markers [12,14], several strain specific DNA sequence variants in the mitochondrial COI gene [15,16] and ND1 gene [17], AFLP markers [18,19], and the FR repetitive nuclear DNA sequence extensively present in R and mostly absent from C [14,20]. Co-occurrence of typically strain-specific markers in the same individuals has provided evidence of some naturally-occurring hybridization in the field [18].

Given that natural hybridization can occur, factors that might limit it and thus maintain the genetic integrity of two different strains are of interest. The two strains differ in the timing of their mating activity; female calling (emission of pheromone) and mating occurs early in the night for C and during the last half of the night for R [21,22]. When using live females as lures in pheromone traps and typing a subset of males caught in these traps, significantly more C males were caught in traps with C females, and R females attracted more R males [21]. Under laboratory conditions, we have found that both strains mate assortatively to some degree as well (G. Schöfl, A. Dill, A.T. Groot, unpubl. res.), which would inhibit hybridization and enhance divergence between the strains.

The female sex pheromone composition and male attraction in the field have been studied in several regions within the North and South American range of *S. frugiperda *(see Table 1). The female pheromone glands were found to contain Z9-14:Ac as the major compound (to which we will refer as M), comprising up to 83%, as well as the second most abundant compound Z11-16:Ac (m), and a number of compounds present in low amounts, such as Z9-12:Ac and Z7-12:Ac (see Table 1) [23-25]. In addition, Brazilian *S. frugiperda *females produce E7-12:Ac [25], a compound not found in other populations so far.

**Table 1:** Means and Coefficients of variation (CV) of each compound when 7 compounds and when 4 compounds of the sex pheromone gland of *Spodoptera frugiperda *are considered.

	Corn – Sc^1^ (n = 17)		Rice – Sc^1^ (n = 22)		Corn^2^ (n = 76)		C × R^2^ (n = 75		R × C^2^ (n = 74)		Rice^2^ (n = 59)	
**7 Compounds**	Mean	CV	Mean	CV	Mean	CV	Mean	CV	Mean	CV	Mean	CV

Z11-16:Ac (m)	12.4	50	7.3	50	12.5	33	13.9	33	10.0	24	9.3	33
Z9-14:Ac (M)	81.8	7	82.4	8	83.5	5.5	82.3	6	86.1	5	84.0	5
14:Ac (a)	1.1	65	1.1	59	1.0	117	0.9	52	0.9	113	1.5	62
Z11-14:Ac (b)	1.1	35	1.6	55	1.0	69	1.0	40	1.0	61	1.5	41
12:Ac (c)	1.1	42	2.0	44	0.4	53	0.5	51	0.7	180	1.0	81
Z9-12:Ac (d)	0.8	52	2.1	55	0.7	89	0.7	77	0.6	70	1.5	86
Z7-12:Ac (e)	1.8	48	3.6	47	0.9	45	0.8	54	0.7	37	1.1	51

**4 Compounds**												

Z11-16:Ac (m)	12.8	50	7.7	51	12.8	33	14.3	33	10.3	25	9.7	33
Z9-14:Ac (M)	84.6	7	86.3	6	85.6	5	84.2	5	88.3	3	87.6	4
Z9-12:Ac (d)	0.8	53	2.3	58	0.7	97	0.7	78	0.6	77	1.6	89
Z7-12:Ac (e)	1.8	49	3.8	48	1.0	45	0.8	54	0.8	39	1.1	53

^1^Glands extracted in the scotophase; ^2^Glands extracted from PBAN-injected females

Not all compounds that are found in the sex pheromone glands of females are "pheromone components" which are by definition important in the attraction of conspecific males (e.g. [6]; some may be unavoidable by-products of the pheromone biosynthetic pathways [4]. The components that have been found to be attractive for *S. frugiperda *males include the major component Z9-14:Ac (M), as well as one of the components present in only low amounts, Z7-12:Ac (to which we refer as e). This is the crucial secondary pheromone component of *S. frugiperda*, because blends without it are not attractive for males of this species [23-29]. The addition of other components has given different results in different regions, which suggests that the sexual communication of this species varies geographically [25], similar to what has been found in several other noctuid moth species (e.g., [30-32]. Specifically, when Z11-16:Ac (m) was added to the blend, significantly more males were attracted in Mexico and Costa Rica [26,27], but not in Florida [23]. In Pennsylvania, adding Z11-16:Ac (m) as well as Z9-12:Ac (d) to the two-component blend attracted twice as many males as the two-component blend [29]. By contrast, in Brazil the addition of Z11-16:Ac to the two-component blend did not increase attraction of *S. frugiperda *males, but the addition of E7-12:Ac, that was found uniquely in glands of Brazilian females, did [25].

Surprisingly, none of the above-described studies mention whether corn or rice strain females were analyzed or whether corn or rice strain males were attracted to the different blends. This is despite the documentation of both strains in North America [12,13,15,21] as well as in Brazil [33]. Because of the lack of distinction between the two strains in previous pheromone studies, the variation found in the different regions could be at least partly due to sampling of two different strains in the different areas.

Another possible source of variation found in the pheromone composition is the time of day at which glands are extracted. Female moths usually produce pheromone *de novo *every night [34], and in many species the timing of pheromone synthesis and release is controlled by Pheromone Biosynthesis Activating Neuropeptide (PBAN) [35]. Extraction of glands after dark (i.e. in the scotophase) yields those compounds that have accumulated there in response to natural PBAN produced in the suboesophagal ganglion and released from the corpus cardiacum into the hemolymph [36,37]. Experimental injection of commercially available PBAN induces pheromone production within 2–3 hours, independent of the time of day or the physiological state (mating status and age) of the female [35,38-41]. This thus can exclude the time of day as a possible source of variation, which is important in the two strains of *S. frugiperda*, because they differ in their time of mating activity and thus likely also the time of pheromone production at night. Previously, we found that injecting females with PBAN can be a simple and convenient method of determining a female's native pheromone phenotype [42]. However, PBAN can act at different stages in the pheromone biosynthetic pathway in different species, reviewed by [4,41]. Therefore, it is important to assess the effect of PBAN injections on the pheromone composition in the two strains.

The aim of our study was to determine whether the pheromone composition in the glands of corn strain females differs from that of rice strain females. We analyzed the pheromone composition of F_1 _hybrid females as well, to assess the mode of inheritance of pheromone variation. We found signficant differences in pheromone composition between corn and rice strain females. Although PBAN reduced the variation in both strains, the pheromone composition remained significantly different. The relative amounts of the two most abundant components, Z9-14:Ac (M) and Z11-16:Ac (m), were maternally inherited, while three minor compounds were inherited in a manner indicating genetic dominance of alleles from the corn strain. Linking these differences to the phenotypic correlations between the pheromone compounds, as well as to a hypothetical scheme of the biosynthetic pathway of the pheromone of this species, suggests that differential activity of a Δ11-, Δ9-, and/or possibly also a Δ7-desaturase may underly the pheromone variation between the two strains.

## Results

### Between-strain pheromone variation

**Figure 1:**
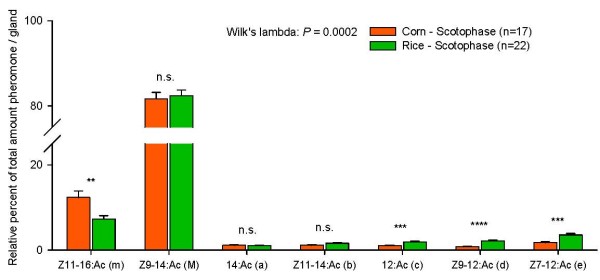
**Between-strain comparisons of the pheromone composition when glands were extracted from females under natural conditions, i.e. 4–6 h into scotophase**. The total percent of all depicted compounds add to 100%. N.s: not significant, * indicates *P *< 0.05, ** indicates *P *< 0.01, *** indicates *P *< 0.001, **** indicates *P *< 0.0001.

Comparing the gland content between corn and rice strain females that had been extracted under natural conditions in the scotophase, we found a significant overall difference in the pheromone composition as determined by the relative amounts of most of the minor compounds (Fig. 1; see Table 1 for Coefficients of Variation). Corn strain females contained significantly more Z11-16:Ac (m) and significantly less 12:Ac (c), Z9-12:Ac (d), and Z7-12:Ac (e) than rice strain females. The relative amount of the major component Z9-14:Ac (M) was the same in the two strains, as well as the relative amount of 14:Ac (a) and Z11-14:Ac (b).

### Effect of PBAN on pheromone composition

**Figure 2:**
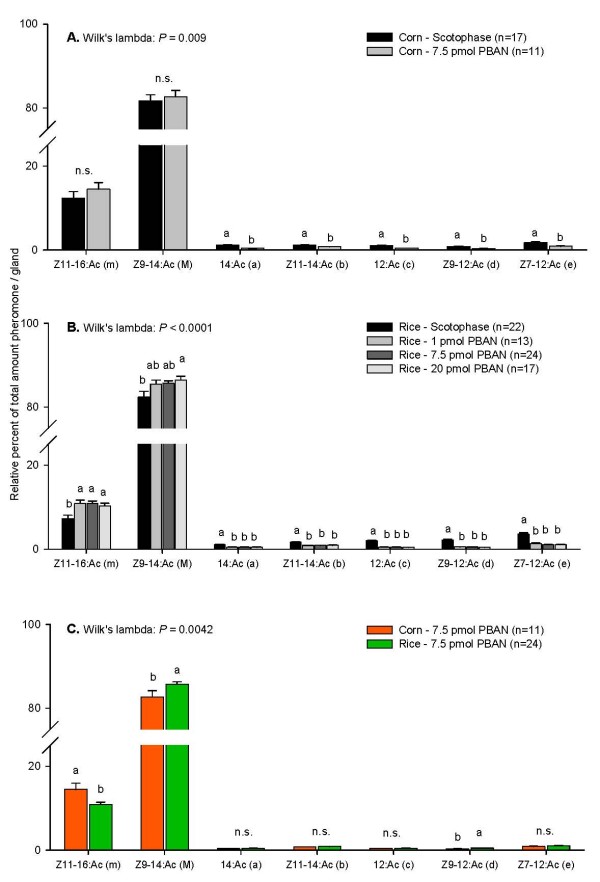
**Effect of PBAN on the pheromone composition in the females of the two strains**. Glands that were extracted from females in the scotophase were compared to glands that were extracted from females that had been injected with PBAN. A. Glands from corn strain females. B. Glands from rice strain females. C. Comparsion between corn and rice strain glands that were extracted from females that had been injected with 7.5 pmol PBAN. In each graph the total percent of all depicted compounds add to 100%. Different letters above the bars of one component indicate significant differences. N.s: not significant.

In both strains, glands of females injected with PBAN differed significantly in their pheromone composition from glands of females that were extracted in the scotophase (Fig. 2a and 2b). In both strains, significantly more Z11-16:Ac (m) and significantly less of all other minor compounds (a-e) were found in glands of PBAN-injected females. There were no differences between the glands of rice strain females injected with different amounts of PBAN (1, 7.5 or 20 pmol) (Fig. 2b). Despite the increase of Z11-16:Ac (m) and a reduction of most compounds in both strains, the overall pheromone composition still differed significantly between the two strains after injection with 7.5 pmol PBAN (Fig. 2c). Specifically, there was still significantly more Z11-16:Ac (m) and significantly less Z9-12:Ac (d) in corn strain than in rice strain females, although differences in the relative amounts of 12:Ac (c) and Z7-12:Ac (e) were no longer significant.

### Mode of inheritance of pheromone variation

**Figure 3:**
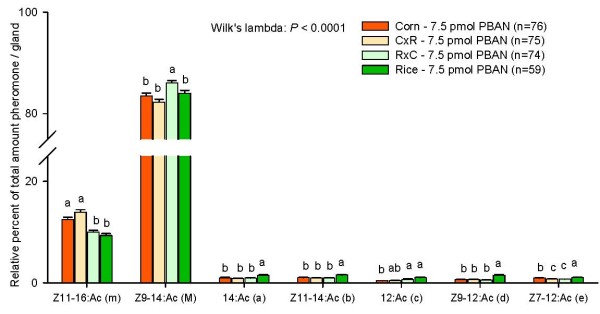
**Mode of inheritance of the pheromone composition**. Gland extracts from offspring of corn females mated with rice males (CxR) and offspring of rice females mated with corn males (RxC) were compared with gland extracts from corn and rice strain females. All females have been injected with 7.5 pmol PBAN before gland extractions. The total percent of all depicted compounds add to 100%. Different letters above the bars of one component indicate significant differences. N.s: not significant.

The hybrid CxR females (offspring of C females and R males) contained similar amounts of the major component Z9-14:Ac (M) as the corn and the rice strain females, while the reciprocal cross RxC females contained significantly more of this component than the other three groups (Fig. 3; see Table 1 for Coefficients of Variation). CxR females contained similar relative amounts of Z11-16:Ac (m) as corn strain females, while RxC females contained similar relative amounts of Z11-16:Ac (m) as rice strain females. This suggests a maternal inheritance of the relative amount of this component. All other compounds were present in similar amounts in both CxR females and RxC females. For 14:Ac (a), Z11-14:Ac (b), and Z9-12:Ac (d), the relative amounts in the hybrids were similar to corn strain females and significantly lower than rice strain females, suggesting genetic dominance of alleles from the corn strain in the production of these compounds in the hybrids. The remaining two compounds 12:Ac (c) and Z7-12:Ac (e) showed a different pattern: RxC females contained significantly more 12:Ac than corn strain females, and both hybrids contained significantly less Z7-12:Ac than the two parental strains.

**Figure 4:**
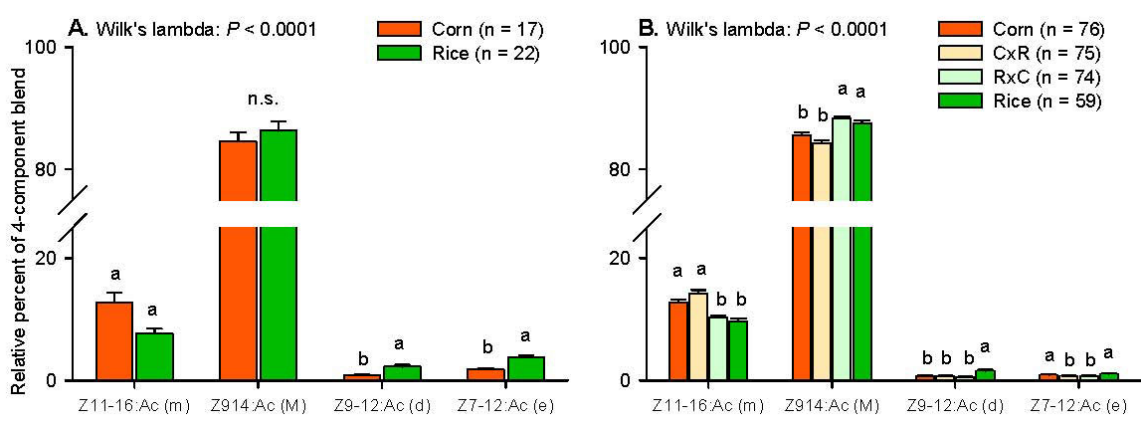
**Between-strain comparisons of the pheromone composition between corn and rice strain females, including only the four compounds that have been shown to affect conspecific male attraction**. The total percent of the four compounds add to 100%. Different letters above the bars of one component indicate significant differences. N.s: not significant.

Not all compounds that we identified from the female glands may be pheromone components functioning to attract males. From the field studies conducted so far, at least the four components Z9-14:Ac (M), Z11-16:Ac (m), Z9-12:Ac (d), and Z7-12:Ac (e) have been shown to affect the attraction of *S. frugiperda *males in one or more populations (see Table 2). The possible attractive role of the other compounds remains to be investigated. When we omitted these other compounds and based our analysis only on the four components that are known to affect the attraction of conspecific males (i.e. the total amount of these four components was set to 100%, after which the relative percentages of each of the four components were recalculated), we also found a significant overall difference in pheromone composition between the corn and the rice strain (see Fig. 4). Specifically, corn strain females contained significantly more Z11-16:Ac (m) and significantly less Z9-12:Ac (d) than rice strain females in all comparisons. The major component Z9-14:Ac (M) was only significantly different between corn and rice strain females when injected with 7.5 pmol PBAN (Fig. 4b). The hybrid females contained similar relative amounts of Z9-14:Ac (M) and Z11-16:Ac (m) as their mothers, indicating a maternal inheritance of the relative amount these main components. Hybrid females of both types contained similar amounts of Z9-12:Ac (d) as corn strain females, similar to what was found when including all pheromone compounds (Fig. 3). The critical secondary sex pheromone component Z7-12:Ac (e) was significantly higher in scotophase-extracted rice strain females than in corn strain females, but did not differ between PBAN-injected corn and rice strain females. This compound was significantly lower in hybrid females.

**Table 2:** Reported sex pheromone components of *S. frugiperda*

Female sex pheromone						Synthetic lures that attracted most males					
	Fl, USA^1^	French Guyana^2^	Brazil^3^	Corn*	Rice*	PA, USA^4^	Fl, ** USA^1,5^	Mexico^6^	Costa Rica^7^	French Guyana^2^	Brazil^3^

**Z11-16:Ac (m)**	9	16.66	12.9	**12.4**	**7.3**	17.69		10.3		15.5	
**Z9-14:Ac (M)**	69	73.75	82.8	**81.7**	**82.4**	81.61	99.42	77.8	99.4	83	98
**14:Ac (a)**		0.53		**1.1**	**1.1**						
**Z11-14:Ac (b)**		1.2	1.5	**1.1**	**1.6**						
**12:Ac (c)**		0.43	0.6	**1.1**	**1.9**						
**Z9-12:Ac (d)**	2	0.5	trace	**0.8**	**2.1**	0.25				0.5	
**Z7-12:Ac (e)**	4	1.12	0.8	**1.8**	**3.6**	0.45	0.58	11.9	0.6	1	1
E7-12:Ac			1.2								1
Z9-14:Al	13	3.59									
Z10-14:Ac			0.3								
16:Ac		0.21									
Z11-16:Al	3										

All numbers in one column add to 100%. Female sex pheromone refers to gland extracts. Tumlinson et al. (1986) also analyzed the volatile blend emitted from the glands and found 2.6% Z11-16:Ac, 90.1% Z9-14:Ac, 1.2% Z11-14:Ac, 1.9% 12:Ac, 2.2% Z9-12:Ac, 0.21% 16:Ac and 3.2% Z7-12:Ac. Note that the Z9-14:Al which was found in the gland extracts, was not found in the emitted volatiles. Compounds in bold are the compounds that we could identify in the gland extracts studied here; all of these were found in both strains. Letters behind these compounds are given the facilitate the reading. *Means of the relative amounts that we found in glands extracted from females in the scotophase. ** In FL the same 4-component blend as used by Fleischer et al. (2005) was tested and attracted similar amounts of males as the 2-component blend.^1^[23]; ^2^[24]; ^3^[25]; ^4^[29]; ^5^[28]; ^6^[27]; ^7^[26].

### Phenotypic correlations

**Figure 5:**
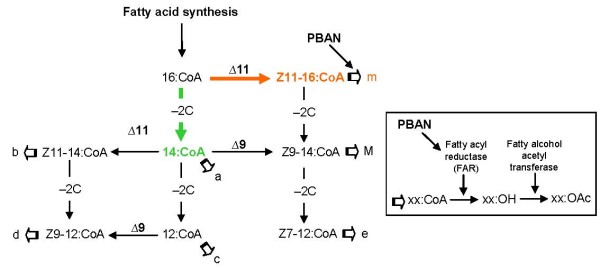
**Proposed pathways of the biosynthesis of the pheromone components in *S. frugiperda *(based on biosynthetic pathways described for other moth species by Jurenka, 2003)**. The interplay of desaturation and chain shortening of 16-, and 14-carbon acyl-CoA derivatives produce mono-unsaturated acyl-CoA precursors that are then reduced and acetylated to produce acetate esters. The number that follows Δ indicates the position of the double bond introduced by the desaturase into the acyl-CoA. -2C indicates chain-shortening by two carbons through β-oxidation. The order of desaturation and chain shortening results in different compounds. The open arrows stand for reduction and acetylation. The letters behind the open arrows stand for the pheromone compounds mentioned in all Tables and Figures.

The previous comparisons have been based on mean proportions of pheromone gland compounds of different groups of females. The proportions within a particular group of females vary slightly from individual to individual around these mean values. The phenotypic correlation matrix describes whether this variation in different components is positively or negatively correlated, or uncorrelated (see Additional files 1, 2, 3). Because the compounds are not produced independently of one another, but are connected due to their biochemical pathways (see Fig. 5), the pattern of correlations may reveal properties of those pathways. A pattern seen within all groups of females tested consisted of strong negative correlations between the major component Z9-14:Ac (M) and both Z11-16:Ac (m) and 14:Ac (a) (orange cells in Additional files 1, 2, 3). Thus, for all groups of females, when more Z9-14:Ac is produced, less Z11-16:Ac and 14:Ac are produced and vice versa. Also, in all groups of females the minor compounds b-e were mostly positively correlated with each other (green cells in Additional files 1, 2, 3). Another pattern seen within all groups of females, except in corn females whose glands were extracted in the scotophase (Additional file 1a), is the strong positive correlations between 14:Ac (a) and the other minor compounds (b-e), and the strong negative correlations between M and b-e (yellow cells in Additional files 1, 2, 3). The most striking difference between PBAN-injected corn females and CxR hybrid females on the one hand, and the other groups of females on the other hand, is the positive correlation between the major component M and the critical secondary sex pheromone component e in the first two groups (blue cell in Additional file 2a and 3a). In scotophase-extracted corn females and in RxC females this correlation is absent, while in the other groups of female this correlation is negative.

## Discussion

The relative amounts of the compounds present in the sex pheromone glands of our laboratory-reared corn and rice strain of *S. frugiperda *were significantly different. Rice strain females contained a significantly lower relative amount of Z11-16:Ac (m) and a correspondingly higher amount of most other minor compounds than corn strain females. Connecting these differences to the hypothetical biosynthetic pathway of these compounds (Fig. 5), the differences between the strains could be explained by a reduced activity of the Δ11 desaturase converting 16:CoA to Z11-16:CoA in the rice strain. This would increase the relative amount of 16:CoA available for chain-shortening to 14:CoA, the precursor of the minor compounds (c-e) that were found in higher relative amounts in the rice than in the corn strain.

The biosynthetic scheme can also account for much of the observed correlation structure within groups of females. It is important to note that since M is the dominant compound, always accounting for more than 80% of the total, modest variations in its production would have proportionally greater effects on the relative amounts of the minor compounds. Also, an increase in the formation of one product would entail a decrease in the formation of the other. The universal and strong negative correlations between the major component Z9-14:Ac (M) and both Z11-16:Ac (m) and 14:Ac (a) are consistent with the assumption that Z11-16:CoA is the precursor for both M and m, and that 14:CoA is the precursor of M and a. Another pattern common to many of the groups is the positive correlation among the four minor compounds b-e, that all share the same common precursor 14:CoA. If the amount of 14:CoA varies between females within a group, then a larger amount could result in a concomitant increase of all products. The significant positive correlation between the major component Z9-14:Ac (M) and the critical secondary sex pheromone component Z7-12:Ac (e), found only in PBAN-injected corn and CxR females, may be explained by an increased conversion of 16:CoA to Z11-16:CoA coupled to a limited availability of fatty acyl reductase (FAR), so that an excess of Z11-16:CoA is chain-shortened (through β-oxidation) to Z9-14:CoA and Z7-12:CoA. Alternatively, PBAN may enhance β-oxidation in the corn strain.

The finding that injection of PBAN changed the ratio of the pheromone compounds in both the corn and the rice strain suggests that PBAN does not primarily stimulate the synthesis of fatty acid hormone precursors, as in most moth species [43-46], but instead primarily stimulates a step later in the biosynthetic pathway, as in *Spodoptera littoralis *[47,48]. In general, the precursors of moth pheromones are derived from the fatty acid synthesis, which are stearic acid (18:CoA) and palmitic acid (16:CoA). These acids can then be desaturated through Δ11- and Δ9-desaturase, and reduced through β-oxidation to shorter chain length fatty acids (e.g. 14:CoA, 12:CoA), after which they are reduced and acetylated to form the acetate esters [49]. In *S. littoralis*, PBAN acts on the reduction step of the fatty acids to form the intermediate alcohols [47,48], which is directly followed by the next enzymatic reaction (acetylation) to form the end products [41]. It seems likely that a similar mode of action of PBAN occurs in *S. frugiperda*: after the formation of Z11-16:CoA, PBAN may activate a fatty acyl reductase (FAR), so that Z11-16:OH is formed, which is then converted to Z11-16:Ac (m) (see Fig. 5). Alternatively, PBAN may specifically activate the Δ11-desaturase that converts the 16:CoA to Z11-16:CoA. This may be a different Δ11-desaturase than the one converting 14:CoA to Z11-14:CoA, similar to the differential substrate preference found for Δ9-desaturases [50]. This would explain the significant increase of Z11-16:Ac (m) in glands of PBAN injected females compared to glands of females that were extracted under natural conditions in the scotophase in both strains (see Fig. 2a and 2b). Despite this effect on the pheromone composition in both strains, decreasing the differences between the two strains, significant differences between the strains were still detectable.

The pheromone composition in hybrid females reveals the mode of inheritance of the pheromone differences between the two strains. The relative amounts of Z11-16:Ac (m) and probably also 12:Ac (c) in the pheromone glands are maternally inherited. In addition, the major component Z9-14:Ac (M) shows a maternal inheritance as well when only the four components are compared that have been shown to affect the attraction of *S. frugiperda *males (see Fig. 4). The other compounds, 14:Ac (a), Z11-14:Ac (b), and Z9-12:Ac (d), are inherited differently, in a corn-dominant way, suggesting that the production of these compounds are affected by a different set of genes. The production of the essential secondary component Z7-12:Ac (e), without which *S. frugiperda *males are not attracted [23], seems to be suppressed in the hybrid females, as both contained less than either parent. We are currently conducting crosses and backcrosses to identify the genetic basis of all pheromone differences between these two strains.

Candidate genes that may possibly underlie the sex pheromone differences between corn and rice strain females can be found by linking these differences to likely enzymatic differences in the biosynthetic pathway (Fig. 5), as well as by comparing the phenotypic correlations between the pheromone compounds. This is a novel approach that we developed recently [51] to generate a list of candidate genes that may explain pheromone differences between species. The most obvious difference in pheromone composition between the two strains of *S. frugiperda*, i.e. a higher relative amount of Z11-16:Ac (m) in corn than in rice strain females, suggests that a Δ11-desaturase, converting the 16:CoA to Z11-16:CoA, is more active in corn than in rice strain females. In addition, the strong positive phenotypic correlation in the rice strain (and RxC females) between 14:Ac (a) and 12:Ac (c) with Z7-12:Ac (e) suggests a coupling that could be explained by a Δ7-desaturase. This coupling is absent in corn females, at least when injected with PBAN. Thus, a Δ7-desaturase, if present, could be restricted to the rice strain. However, so far Δ7-desaturases have not been identified in insects. An alternative explanation is that Z7-12:Ac in rice-strain females is produced via a conversion of 14:CoA by Δ9-desaturase to Z9-14:CoA, after which Z9-14:CoA is β-oxidized to Z7-12:Ac (e). In corn females (and CxR females), the positive correlation beween Z9-14:Ac (M) and Z7-12:Ac (e), and not between M and 14:Ac, suggests that Z7-12:Ac (e) in this strain is produced via Δ11-desaturation of 16:CoA, which is subsequently β-oxidized to Z9-14:Ac (M) and Z7-12:Ac (e), as mentioned above. Thus, both Δ11-desaturase and Δ9-desaturases, and possibly Δ7-desaturase, are candidate genes that may be differentially active between the two strains. Desaturases have been identified from pheromone glands of many moth species, e.g., [52]. In the genus *Spodoptera*, Δ9- and Δ11-desaturases have been characterized in *S. exigua*, *S. littura *[52] and *S. littoralis *[53]. These identifications will facilitate the assessment of whether and which of these genes vary between the two strains.

Now that we have found significant differences in the pheromone composition between the two strains, the next steps are to evaluate a) whether different ratios of the pheromone blend are differentially attractive to corn and rice strain males, and b) how the between-strain variation is related to the geographic variation in sexual communication that has been found in the past. Strain-specific lures will probably be more effective to assess the population distributions of the two strains than the commercial lures that have been used so far, e.g., [27,54-56]. If the ratios found in the glands are an indication of the ratios emitted by the females, a blend with a larger relative amount of Z11-16:Ac is likely to be more attractive to corn than to rice strain males. If Z11-16:Ac is less important in the attraction of rice strain males, this might explain why the addition of this compound to the two-component blend did attract more males in Mexico [27] and Costa Rica [26], but not in Florida [23] or Brazil [25]: the latter experiments may have been conducted in areas or periods when mostly rice-strain males were present.

## Conclusion

The two strains of *S. frugiperda *are not only differentiated in their host use and their timing of sexual activities at night, but also in their sex pheromone composition. We found significant differences in the pheromone blend between the two strains when we considered the seven compounds that were identified from the pheromone gland of this species (see Table 1), and when we only considered the four pheromone components that have been shown to affect the attraction of *S. frugiperda *males. Even when females were injected with PBAN, which reduced the among-strain differences, the pheromone composition still significantly differed between the two strains. The pheromone composition of the hybrid females suggests a maternal inheritance of the relative amount of Z11-16:Ac (m) and a genetic dominance of alleles from the corn strain in the production of 14:Ac (a), Z11-14:Ac (b), and Z9-12:Ac (d). The traits that differentiate the two host strains of this species (host use and differential timing of sexual activities at night) are most likely not independent of each other. For example, host plants have been found to directly or indirectly affect the sexual communication in moths [57-59]. Finding the genetic basis of both sexual (i.e. behavioral) and host plant (i.e. habitat) differentiation in this species will potentially give insight into the interaction between behavioral isolation and habitat isolation, and their role in speciation [60].

## Methods

### Insects

FAW corn and rice strains were obtained in 2006 from lab-reared colonies of R. Meagher at USDA-ARS in Gainesville, Fl. The corn strain was established from > 100 larvae collected from corn plants near Homestead in Miami-Dade Co., Fl, throughout October and November 2004, and was called JS3C. The rice strain colony originated from > 200 larvae collected from pasture grasses from the Range Cattle Research and Education Centre, Ona, Hardee Co., Fl, between May 2003 and October 2003, and was named OnaR. Both colonies were reared in mass culture for 10 and 21 generations, respectively, on a pinto bean-based artificial diet at USDA Florida. In July 2006, a subset of these colonies were transferred to our laboratory. Upon receiving the colonies, 48 JS3C and 56 OnaR individuals were screened for the strain-specific COI marker. All but three JS3C individuals had the RFLP marker that is associated with the corn strain, and all OnaR individuals had the RFLP marker that is associated with the rice strain [15,16]. Offspring of the three ambiguous JS3C individuals were not included in subsequent rearing. Individuals used for our experiments had been reared for another 15 generations in our laboratory, in environmental chambers at 26 ± 1°C, 60 ± 10% RH, and a 14:10 L:D photoperiod, in a single pair mating protocol that is specifically designed to maintain the genetic variation and to avoid selection of any females. In short, in every generation an equal number of offspring is chosen from each of 30 single pair matings. The offspring from these matings are randomly paired, again in single pairs, in the next generation to maximize effective population size and avoid possible shifts in allele frequencies. Adults used for the pheromone experiments were placed as larvae in a reversed L:D chamber, where lights were off from 11.00 – 21.00 h. Pupae were kept individually in plastic cups and checked daily for emergences. Emerged females were given a honey-water solution and left in the same rearing chamber until their glands were extracted. Pheromone glands were extracted from 2–3 day old virgin females.

### Gland extractions

Two groups of glands were extracted, one to assess the between-strain variation and mode of inheritance of strain differences (Group I), and one to assess the effect of PBAN (Group II). Of group I, glands were extracted from corn and rice strain females, as well as from hybrid female offspring from crosses with corn females and rice males, referred to as CxR, and from crosses with rice females and corn males, referred to as RxC. To minimize the effect of one specific cross, glands were extracted from offspring of 9–10 crosses. All females were injected with Hez-PBAN (Peninsula Laboratories, San Carlos, CA) ca. 1–2 h before the scotophase. A stock solution of *Hez*-PBAN (200 pmol/*μ*l in 50% methanol and 1 N HCl) was diluted in saline to 3.75 pmol/ul within 1 hr of injection. Females were injected with 2 *μ*l of this dilution, using a 10 *μ*l syringe (Hamilton, Reno, NV) with a 31 gauge needle that was inserted ventrally between the 8th and the 9th abdominal segments. All females were injected at similar times, 1–2 h before the scotophase, and glands were dissected 2–3 hours later. Of group II, glands were extracted either from females 4–6 h into scotophase, or from females that were injected with PBAN ca. 1–2 h before the scotophase. To assess whether possible changes in the pheromone composition was dependent on the dose of PBAN injected, rice females were injected with 2 ul of PBAN of either 0.5, 3.75 or 10 pmol/ul. Because of a limited number of corn females available at the time of this experiment, corn females were injected only with 2 ul of 3.75 pmol/ul (the second dilution). Two to three hours after PBAN injection, the glands were dissected from the females.

All pheromone glands were dissected and placed in conical vials containing 50 *μ*l hexane and 40 ng pentadecane as internal standard. After 30–40 min, the glands were removed and the extracts were stored at -20°C until analysis. The hexane extract was reduced under a gentle stream of N_2 _to 1–2 μl, taken up into 2 μl octane, and placed in a 50 μl glass insert within a crimp-capped vial. Using a 7683 automatic injector, the entire volume (i.e. 3–4 μl) of extract was injected into a splitless inlet of a HP7890 gas chromatograph (GC) coupled with a high resolution polar capillary column (DB-WAXetr [extended temperature range]; 30 m × 0.25 mm × 0.5 μm) and a flame-ionization detector (FID), programmed from 60°C with a 2 min hold, to 180°C at 30°C/min, then to 230°C at 5°C/min, during which all the pheromone components eluted. The column was then heated to 245°C at 20°C/min and held at this temperature for 15 min to clean the column before the next analysis. The FID detector was held at 250°C.

### Chemical analysis

Of all the compounds that have been previously identified from glands of *S. frugiperda *(see Table 1), we identified the following from the pheromone glands: Z9-14:Ac (M), Z11-16:Ac (m), 14:Ac (a), Z11-14:Ac (b), 12:Ac (c), Z9-12:Ac (d), and Z7-12:Ac (e). The letters behind the compound are given to facilitate the reading. All of these compounds were found in both strains. These compounds were first identified by injecting synthetic compounds into the GC described above to compare their retention times with the retention times of the peaks present in the gland extracts. In addition, the alcohol and some of the aldehyde analogs of these compounds (i.e., Z9-14:Ald, Z11-16:Ald, Z9-12:OH and Z7-12:Ac) were injected as well to make sure that these compounds have a different retention time in our settings and GC program. All synthetic compounds were bought from Pherobank, Wageningen. After the first identification, the chemical identities of all peaks in the gland extracts were checked by GC-MS. A subset of extracts was injected into an HP6890 GC coupled to Masspec MS002 (Micromass, Manchester, UK) with electron ionization (EI) at 70 eV, and separated using a 30 m × 0.25 mm × 0.25 μm DB-Wax column, with the same temperature program as described above. The recorded mass spectra were compared to those of known standards injected in the same manner and using spectral database (Wiley MS library v 7). The gland extracts were additionally screened for a possible detection of E7-12:Ac. This compound was not found in the 10 extracts examined.

The amount of each pheromone compound was calculated relative to the 40 ng internal standard. Every day, before and after each GC sequence, we injected authentic standards of the pheromone compounds mentioned above to assess column performance as well as to check the retention times of each of the components. We corrected all integration results by the differential response of the FID to the various authentic standards. Because there is high variance among female moths in total gland pheromone content, even within treatments, most researchers analyze differences between the amount of each component after converting amounts to percentages relative to the most abundant compound (i.e., the "major" component) (e.g. [8,23]. Therefore, we compared the pheromone composition between different groups of females by converting all amounts to relative percentages of the total amount of all pheromone components in the glands.

### Statistical analysis

To compare the pheromone compositions between the two strains, a multivariate analysis of variance (MANOVA) was conducted, using SAS, version 9.1 (SAS Institute, 2002–2003), with all the females of a group that were a) extracted during the scotophase, or b) injected with 7.5 pmol PBAN. In addition, to assess the effect of PBAN on the pheromone composition within each strain, a separate MANOVA was conducted within each species. To determine the mode of inheritance of the difference in pheromone composition between corn and rice strain females, another MANOVA was conducted, comparing the pheromone composition between the corn strain females, the rice strain females, the CxR hybrid females, and the RxC hybrid females. In every test, the means of all pheromone compounds were separated using least-squares means (LSMEANS), with a Tukey adjustment for multiple comparisons.

### Phenotypic correlations

To assess possible enzymatic differences between the two strains in the biosynthetic pathway of the FAW pheromone, we analyzed phenotypic associations among pheromone components in the two strains by generating a Pearson's correlation matrix (PROC CORR in SAS). For this correlation matrix we used the percentages of each compound relative to the total amount of all the pheromone compounds that we distinguished. Separate correlation matrices were constructed for the pheromone composition found in glands of a) corn or rice females from which glands were extracted under natural conditions, i.e. in the scotophase, b) corn or rice females that were injected with 7.5 pmol PBAN, which glands were extracted at the same time as the hybrid females, and c) hybrid CxR or RxC females that were injected with 7.5 pmol PBAN as well. Since such associations can give insight into biosynthetic pathways of the pheromone components, we also constructed a (hypothetical) scheme of this pathway.

## Abbreviations

The chemical compounds are abbreviated following the standard shorthand notation for pheromone molecules [61]. For example, (*Z*)-11-hexadecen-1-yl acetate is abbreviated Z11-16:Ac. The corresponding aldehyde is abbreviated Z11-16:Al. PBAN: Pheromone Biosynthesis Activating Neuropeptide.

## Competing interests

The authors declare that they have no competing interests.

## Authors' contributions

ATG designed the experiments. MM conducted the experiments. ATG, MM and GS analyzed the data. SL and AS carried out the chemical analyzes and identifications. ATG and DGH interpreted the data and drafted the manuscript. MM, GS, SL and AS critically revised the manuscript. All authors read and approved the final manuscript.

## Supplementary Material

Additional file 1 Pearson's correlation coefficients of pheromone compounds in glands of A) corn strain females, and B) rice strain females, extracted in scotophase. The tables show positive and negative phenotypic correlations between all pheromone compounds. The colors of the cells coincide with the colors in the proposed biosynthetic pathway of the compounds in Figure 5. Click here for file

Additional file 2 Pearson's correlation coefficients of pheromone compounds in glands of A) PBAN-injected corn strain females, and B) PBAN-injected rice strain females. The tables show positive and negative phenotypic correlations between all pheromone compounds. The colors of the cells coincide with the colors in the proposed biosynthetic pathway of the compounds in Figure 5, while the blue cells indicate a strikingly different correlation from that found in other females. Click here for file

Additional file 3 Pearson's correlation coefficients of the pheromone compounds in glands of A) CxR hybrid females, and B) RxC hybrid females. The tables show positive and negative phenotypic correlations between all pheromone compounds. The colors of the cells coincide with the colors in the proposed biosynthetic pathway of the compounds in Figure 5, while the blue cell indicates a strikingly different correlation from that found in other females. Click here for file
