# Supplementary material for: Host strain specific sex pheromone variation in Spodoptera frugiperda
Source: Front Zool. 2008 Dec 25;5:20. doi: 10.1186/1742-9994-5-20 (PMC2628650; doi:10.1186/1742-9994-5-20)
Supplement: Additional file 2 — Pearson's correlation coefficients of pheromone compounds in glands of A) PBAN-injected corn strain females, and B) PBAN-injected rice strain females. The tables show positive and negative phenotypic correlations between all pheromone compounds. The colors of the cells coincide with the colors in the proposed biosynthetic pathway of the compounds in Figure 5, while the blue cells indicate a strikingly different correlation from that found in other females. [file 1742-9994-5-20-S2.doc]

| A. Corn (n = 76) | Z11-16:Ac  (**m**) | Z9-14:Ac  (**M**) | 14:Ac  (**a**) | Z11-14:Ac  (**b**) | 12:Ac  (**c**) | Z9-12:Ac  (**d**) |
| --- | --- | --- | --- | --- | --- | --- |
| Z9-14:Ac (**M**) | **-0.86****** | — |  |  |  |  |
| 14:Ac (**a**) | -0.05 | **-0.44****** | — |  |  |  |
| Z11-14:Ac (**b**) | 0.15 | **-0.53****** | **0.71****** | — |  |  |
| 12:Ac (**c**) | 0.07 | **-0.27*** | **0.34**** | 0.05 | — |  |
| Z9-12:Ac (**d**) | -0.19 | **-0.29*** | **0.82****** | **0.63****** | **0.39***** | — |
| Z7-12:Ac (**e**) | **-0.37**** | **0.27*** | 0.02 | **-0.24*** | 0.07 | 0.03 |

| B. Rice (n = 59) | Z11-16:Ac  (**m**) | Z9-14:Ac  (**M**) | 14:Ac  (**a**) | Z11-14:Ac  (**b**) | 12:Ac  (**c**) | Z9-12:Ac  (**d**) |
| --- | --- | --- | --- | --- | --- | --- |
| Z9-14:Ac (**M**) | **-0.61****** | — |  |  |  |  |
| 14:Ac (**a**) | 0.03 | **-0.67****** | — |  |  |  |
| Z11-14:Ac (**b**) | 0.09 | **-0.57****** | **0.73****** | — |  |  |
| 12:Ac (**c**) | -0.03 | **-0.59****** | **0.45***** | **0.26*** | — |  |
| Z9-12:Ac (**d**) | **-0.28*** | **-0.53****** | **0.58****** | **0.32*** | **0.69****** | — |
| Z7-12:Ac (**e**) | **-0.40**** | **-0.31*** | **0.38**** | **0.28*** | **0.46***** | **0.84****** |

Females were injected with 7.5 pmol PBAN, at the same time as the hybrid females (Group 1).

The sum of all components is set to 100%. Significant interactions are shown in bold.

* indicates *P* < 0.05, ** indicates *P* < 0.01, *** indicates *P* < 0.001, **** indicates *P* < 0.0001
